# miRNA-132-5p mediates a negative feedback regulation of IL-8 secretion through S100A8/A9 downregulation in neutrophil-like HL-60 cells

**DOI:** 10.3389/fimmu.2023.1274378

**Published:** 2024-01-16

**Authors:** Yang Zhou, Milène Tetsi Nomigni, Anthoula Gaigneaux, Fabrice Tolle, Helen L. Wright, Jean-Luc Bueb, Sabrina Bréchard

**Affiliations:** ^1^ Department of Life Sciences and Medicine, University of Luxembourg, Esch-sur-Alzette, Luxembourg; ^2^ Institute of Life Course and Medical Sciences, University of Liverpool, Liverpool, United Kingdom

**Keywords:** neutrophils, cytokines, Ca^2+^ signaling, S100A8/A9, miRNA, inflammation

## Abstract

**Background:**

Neutrophils are an important source of pro-inflammatory and immunomodulatory cytokines. This makes neutrophils efficient drivers of interactions with immune and non-immune cells to maintain homeostasis and modulate the inflammatory process by notably regulating the release of cytokines. Ca^2+^-dependent regulatory mechanism encompassing cytokine secretion by neutrophils are not still identified. In this context, we propose to define new insights on the role of Ca^2+^-binding proteins S100A8/A9 and on the regulatory role of miRNA-132-5p, which was identified as a regulator of S100A8/A9 expression, on IL-8 secretion.

**Methods:**

Differentiated HL-60 cells, a human promyelocytic leukemia cell line that can be induced to differentiate into neutrophil-like cells, were used as a model of human neutrophils and treated with N- formyl-methionyl-leucyl-phenylalanine (fMLF), a bacterial peptide that activates neutrophils. shRNA knockdown was used to define the role of selected targets (S100A8/A9 and miRNA-132-5p) on IL-8 secretion.

**Results and discussion:**

Different types of cytokines engage different signaling pathways in the secretion process. IL-8 release is tightly regulated by Ca^2+^ binding proteins S100A8/A9. miRNA-132-5p is up-regulated over time upon fMLF stimulation and decreases S100A8/A9 expression and IL-8 secretion.

**Conclusion:**

These findings reveal a novel regulatory loop involving S100A8/A9 and miRNA-132-5p that modulates IL-8 secretion by neutrophils in inflammatory conditions. This loop could be a potential target for therapeutic intervention in inflammatory diseases.

## Introduction

Over the past few years, neutrophils have gained considerable importance in light of their contribution to fine-tuning inflammatory and immune responses. To fulfill such functions, neutrophils are able to ensure a *de novo* cytokine production, dependent on gene induction, followed by their release according to microenvironmental conditions. Secretion of these soluble messengers allows neutrophils to exhibit remarkable flexibility and modulate innate and adaptive immunity (1). Besides *de novo* synthesis, neutrophils also have the capacity in a resting state to express cytokines from preformed stores (2). Until now, the types of cytokines *de novo* synthesized or/and packaged in intracellular storage sites are still not identified. One of the rare consensus on neutrophil cytokines is that multiple secretory pathways coexist allowing the release of diverse pro-inflammatory mediators (3). Given the complexity of signaling pathways initiated by the engagement of different categories of receptors and the difficulty of genetically manipulating neutrophils, a huge gap persists in the understanding of molecular mechanisms underlining cytokine mobilization in neutrophils.

Two main classical secretion pathways have been identified through which cytokines can be secreted. The current prevailing idea is that preformed cytokines, packaged into granules or secretory vesicles, are instantaneously released during the “regulated exocytosis” process following ligand-mediated receptor activation (3, 4) while *de novo* synthesized cytokines are liberated to the extracellular environment via the “constitutive exocytosis” after their transit between the trans-Golgi network and trafficking through recycling endosomes (5, 6).

In order to characterize the signaling pathways involved in cytokine secretion, the role of Ca^2+^ mobilization has been tackled in a few studies. Extracellular Ca^2+^ entry was described to have a central role in the release of cytokines in human and murine neutrophils subjected to different types of pro-inflammatory stimuli (7, 8). In mice, STIM proteins, key components of the so-called “store-operated Ca^2+^ entry” mechanism (SOCE) (9), by finely sensing Ca^2+^ levels within the endoplasmic reticulum (ER), were claimed to be essential regulators of cytokine secretion. STIM2 appears to be the predominant isoform involved in this process with an exclusion of a role for STIM1 (7, 8). However, other actors intervening in Ca^2+^-dependent signaling pathways are still unknown. Our recent work (10) underlined the potential importance of S100A8/A9 in cytokine secretion in mouse and human neutrophil cell models treated with lipopolysaccharides (LPS).

S100A8/A9 belong to the S100 superfamily of Ca^2+^-binding proteins, which is characterized by an EF-hand motif transducing Ca^2+^ signals to target proteins (11). The S100A8/A9 heterodimeric complex represents up to 40% of the cytosolic protein content in neutrophils. Binding of Ca^2+^ allows the conformational change of S100A8/A9 heterodimer into a heterotetrametric S100A8/A9 complex (12, 13). This latter form is considered as the privileged form for the intracellular biological functions of S100A8/A9 (14). These proteins have long been considered for their dual extracellular role. Indeed, on the one hand, secreted S100A8/A9 (15) can exert anti-inflammatory effects through the suppression of cytokine secretion and scavenging of reactive oxygen species (ROS) released from activated leukocytes (16–18). On the other hand, extracellular S100A8/A9 are considered as damage-associated molecular patterns (DAMPs) and can act as danger signals. In this sense, S100A8/A9 can increase cytokine secretion in immune and non-immune cells (19–21) and exert high chemotactic activities on diverse cell types (22).

Intracellularly, S100A8/A9 can regulate neutrophil migration by contributing to the rearrangement of the cytoskeleton (23) and heighten ROS production through the delivery of arachidonic acid NADPH oxidase subunits (24) or through p38 MAPK activation (25).

Our objective in this study is to define whether S100A8/A9 can be a central effector of the regulation of cytokine secretion and how S100A8/A9-mediated signaling pathways can be regulated in order to prevent an excessive or uncontrolled amplification of the inflammatory response. For that, we identified miRNAs potentially involved in the regulation of S100A8/A9 expression since miRNAs primarily regulate gene expression via post-transcriptional repression (26). Based on bioinformatics analysis, miRNA-132-5p was predicted to regulate S100A8/A9 expression. Overexpression of miR-132-5p was able to downregulate S100A8/A9 expression resulting in a decrease of IL-8 secretion.

A few specific miRNAs have been previously reported to be involved in neutrophil proinflammatory functions. miR-223 knockout in mice resulted in a high susceptibility to bacterial infection and spontaneous development of inflammatory lung pathology characterized by hypersensitive neutrophils to TLR4 stimulation (27). Besides miR-223, miR-146a and miR-155 can suppress the expression of pro-inflammatory cytokines *via* negative feedback loops, through the targeting of key proteins in the TLR4/NF-κB pathway (28–30). New methods based on engineering neutrophil extracellular vesicles in order to ensure a specific and efficient delivery of miRNAs to target cells or tissues are currently considered as a promising tool for therapeutic applications (31). In this regard, miRNA-223 packaged in neutrophil extracellular vesicles and transferred to target cells has been reported to have an anti-inflammatory role by exerting a synergistic effect on neutrophils and macrophages (32). Therefore, the identification of miRNAs such as miRNA-132-5p, with the capacity to modulate pro-inflammatory neutrophil functions, including cytokine secretion could be useful in the development of novel anti-inflammatory treatments.

## Materials and methods

### Cell culture

The human promyelocytic leukemia HL-60 cell line (33) was purchased from ATCC^®^ (CCL-240™). The culture medium used was RPMI-1640 (Life Technologies) supplemented with 2 mM L-glutamine (Life Technologies), 10% v/v complement heat-inactivated fetal bovine serum (Sigma-Aldrich), 100 µg/mL streptomycin (Life Technologies) and 100 U/mL of penicillin (Life Technologies). Cells were cultured at 37°C in a humidified atmosphere with 5% CO_2_ and were passaged at a density of 1x10^6^/mL. Differentiation towards neutrophil-like cells was performed in fresh complete RPMI 1640 containing DMSO (1.3% v/v) for 4.5 days (34).

### Neutrophil isolation

Peripheral blood from healthy volunteers was collected into EDTA-containing tubes (Vacutainer^®^, BD Biosciences), in collaboration with Ketterthill laboratories (Luxembourg). Samples were processed according to the good clinical and ethical practices, which were approved by the Ethics Review Panel (ERP) of the University of Luxembourg based on the guidelines of the “Comité National d’Ethique de Recherche” (CNER) from Luxembourg and the University of Liverpool Central University Research Ethics Committee D.

Erythrocytes in the samples were removed by using MACSxpress™ Erythrocyte Depletion Kit (Miltenyi Biotec). Neutrophils were purified by using MACSxpress^®^ Whole Blood Neutrophil Isolation Kit (Miltenyi Biotec). All the manipulation procedures were performed according to the manufacturer’s instructions. The purity of isolated neutrophils was assessed by polychromatic flow cytometry (FACSCanto II, BD Biosciences) using the following conjugated antibodies towards specific cell surface markers: CD16-BV421, CD45-PE-Cy7, and CD49d-PE (BD Biosciences). Purified neutrophils were immediately incubated with Live/Dead^®^ Fixable Near-IR Dead Cell Stain Kit (ThermoFisher Scientific), fixed with 3.7% Paraformaldehyde (PFA), blocked with 10% v/v human IgG and further incubated with the mixture of conjugated antibodies. Purity was determined on 20.000 events in the gated population of homogenous (FSC-A *vs.* SSC-A), single (FSC-A *vs.* FSC-H) and living leukocytes (negative for Live/Dead Near-IR staining and positive for CD45). Cell populations with CD16^+^ and CD49d^-^ were considered to be neutrophils. Only preparations with a neutrophil purity of ≥ 99% were used for experiments.

### Intracellular IL-8 staining

Intracellular IL-8 production was measured using flow cytometry (FACSCanto II, BD Biosciences). Two cell surface markers CD71 and CD11b (BD Biosciences), were used to discriminate differentiated HL-60 cells (dHL-60) from undifferentiated cells (35). Cells (2x10^6^ cells/mL) were resuspended in a physiological salt solution (PSS; 115 mM NaCl, 5 mM KCl, 1 mM KH_2_PO_4_, 10 mM D-Glucose, 1 mM MgSO_4_, 1.25 mM CaCl_2_ and 25 mM HEPES, pH 7.4) and transferred into a 24-well plate (Corning) with ultra-low attachment surface before fMLF stimulation. Cells were then collected and incubated with Live/Dead^®^ Fixable Near-IR Dead Cell Stain (ThermoFisher Scientific). FcR on cell surface was blocked by incubating the cells with FACS buffer (137 mM NaCl, 2.6 mM KCl, 8 mM Na_2_HPO_4_, 1.8 mM KH_2_PO_4_, 0.15% w/v BSA) containing 1 μg/μL purified human IgG (Sigma-Aldrich). A mixture of conjugated antibodies containing anti-CD71-FITC (BD Biosciences, clone M-A712) and anti-CD11b-BV421 (BD Biosciences, clone ICRF44) or isotype antibodies IgG1-BV421 (BD Biosciences, clone ICRF44) and IgG2a-FITC (BD Biosciences, clone X39) was added to cells. Cells were then fixed by intracellular fixation buffer (eBioscience) and washed by FACS buffer before permeabilization with 1X permeabilization buffer (eBioscience) containing 1% BSA, and 1 μg/μL purified human IgG (Sigma-Aldrich). Finally, conjugated IL-8-PE antibody (BD Biosciences, clone G265-8) and its isotype IgG2b-PE (eBioscience, clone 27-35) was incubated with the cells and resuspended with FACS buffer for flow cytometry acquisition. Data analysis was performed using FACSDiva software (BD Biosciences) and FlowJo™ v10 Software (BD Biosciences). Median Fluorescence Intensity (MFI) of IL-8 staining was calculated and recorded based on gated population of granulocyte (FSC-A *vs.* SSC-A), single (SSC-A vs. SSC-H) and living cells (negative for Live/Dead™ Fixable Near-IR Dead Cell Stain). For each condition, at least 20.000 events for cells and 1.000 events for beads were recorded during acquisition.

### Cytokine secretion

Secretion of IL-8 in the supernatants of dHL-60 cells was measured using ELISA kits (R&D systems) according to the instructions provided by the manufacturer. To evaluate the non-specific release of cytokines by cell death, cell mortality was analyzed by measuring lactate dehydrogenase (LDH) activity in supernatants of cell culture according to the instructions of CytoTox 96^®^ Non-Radioactive Cytotoxicity Assay (Promega).

NF-κB activation assay - NF-κB (p65) transcription factor activation was measured by using a transcription factor assay kit (TransAM; Active motif, 400096) according to the manufacturer’s protocol. Briefly, nuclear extracts were prepared using a nuclear isolation kit (Cayman Chemicals, 10009277), and were then loaded onto coated wells. After incubation with primary and secondary antibodies, the absorbance of antigen-antibody complex was read at 450 nm using BioTek Cytation 5.

### Total RNA extraction

Total RNA extraction from dHL-60 cells was performed using the Quick-RNA Miniprep Kit (ZYMO Research) according to the manufacturer’s instructions. Total RNA extraction from human neutrophils was performed by Trizol chloroform extraction method as per manufacturer’s instructions. Total RNA was cleaned using the Qiagen miRNeasy kit including an on-column DNAse digest.

### miRNA-sequencing

miRNA-sequencing (ENA, PRJEB64660) was performed by EXIQON after RNA quality control using the 2100 Bioanalyzer (Agilent Technologies). Next generation sequencing (NGS) libraries were prepared, quantified and sequenced using single end reads with the ILLUMINA Nextseq500 instrument. Quality control, alignment and differential expression analysis was provided by EXIQON. Statistical analyses were performed by using the trimmed mean of M-values normalization method (TMM normalization). The volcano plot was generated in R program (version 4.2.1) by using tidyverse packages.

### Network analysis of miRNAs

Network analysis of potential miRNAs regulating S100A8/A9 was performed in R program (version 4.2.1) using data from miRTarBase 8.0 and TargetScan 7.2. The link to the file that was used for analysis is as follows: https://mirtarbase.cuhk.edu.cn/~miRTarBase/miRTarBase_2019/cache/download/8.0/hsa_MTI.xlsx.

### Reverse transcription quantitative real-time PCR

For mRNA measurement, reverse transcription was performed in 9700 GeneAmp thermocycler (Applied Biosystems) by using PrimeScript™ RT Reagent Kit (Takara Bio). All the steps were performed according to the manufacturer’s instructions. Primers for each target and reference gene were designed from published sequences in GenBank (**Table 1**) by using Primer3 online software. qPCR was performed with the SYBR^®^ Select master Mix (ThermoFischer Scientific) in the QuantStudio 12K Flex real-time PCR machine (Applied Biosystems). The following temperature protocol was used: 3 min at 50°C, 3 min at 95°C followed by 40 cycles of 3 s at 95°C, 30 s at 60°C. The relative quantification of mRNAs was normalized by three reference genes (*Actin-β, GUSβ, B2M*) following the Vandesompele method (36).

**Table 1:** List of primers used in this study.

	Gene abbreviation	Forward (5'-3')	Reverse(3'-5')
Taget genes	*IL-1A*	*GGAGATGCCTGAGATACCCA*	*CCGTGAGTTTCCCAGAAGAA*
	*IL-1B*	*CACATGGGATAACGAGGCTT*	*TCCATATCCTGTCCCTGGAG*
	*TNF-a*	*GGAGCCAGCTCCCTCTATTT*	*GGCTACATGGGAACAGCCTA*
	*CCL5*	*CCATATTCCTCGGACACCAC*	*ACACACTTGGCGGTTCTTTC*
	*CCL2*	*CCCCAGTCACCTGCTGTTAT*	*TGGAATCCTGAACCCACTTC*
	*CCL3*	*TCTGCAACCAGGTCCTCTCT*	*TTTCTGGACCCACTCCTCAC*
	*CCL4*	*CTTCCTCGCAACTTTGTGGT*	*GCTCAGTTCAGTTCCAGGTCA*
	*IL-8*	*GCAGAGGGTTGTGGAGAAGT*	*CATCTGGCAACCCTACAACA*
	*IL-6*	*CCTGCAGAAAAAGGCAAAGA*	*AAAGCTGCGCAGAATGAGAT*
	*S100A8*	*TCATCGACGTCTACCACAAGT*	*CCAACTCTTTGAACCAGACG*
	*S100A9*	*GGGAATTCAAAGAGCTGGTG*	*GCTGCTTGTCTGCATTIGTG*
	*NF-KB3*	*CCCCAACTTTGTGGATGTCT*	*ACAGAGAAGGGAGCTGACCA*
Reference genes	*Actβ*	*GCCCTGAGGCACTCTTCCA*	*TGTTGGCGTACAGGTCTTTGC*
	*Gusβ*	*CAAGAGCCAGTTCCTCATCA*	*TTGAAGTCCTTCACCAGCAG*
	*B2M*	*AAGCAGCATCATGGAGGTTT*	*TGGAGCAACCTGCTCAGATA*

For miRNA measurement, first-strand cDNA synthesis was performed using miRCURY LNA RT kit (Qiagen) according to the manufacturer’s instructions. Primers for miR-132-3p (Qiagen, YP00206035), miR-132-5p (Qiagen, YP00204552) as well as for reference genes SNORD44 (Qiagen, YP00203902), miR-103a-3p (Qiagen, YP00204063), and SNORD38B (Qiagen, YP00203901) were purchased from Qiagen. qPCR was performed using miRCURY LNA SYBR^®^ Green PCR kits (Qiagen) in a QuantStudio 12K Flex real-time PCR machine. The thermal cycling protocol was: 2 min at 95°C followed by 40 cycles of 10 s at 95°C and 60 s at 56°C. Relative expression of miRNAs was normalized against the three reference genes according to the Vandesompele method (36).

### Western blot

For cytosol protein preparations, dHL-60 cells were lysed in a fresh Triton lysis buffer (50 mM Tris pH 8.0, 150 mM NaCl, 1% Triton) supplemented with protease inhibitor cocktails (SigmaFAST Protease Inhibitor Cocktail Tablet, EDTA free). The protein concentration was determined using Pierce BCA Protein Assay Kit (ThermoFisher Scientific). Loading buffer (63 mM Tris pH 6.8, 2% SDS, 10% Glycerol, 1% β-mercaptoethanol) was added to protein samples and then heated for 10 min at 96°C. Proteins were run on a Tris-Tricine gel (10% acrylamide). Proteins were electrotransferred to a PVDF membrane (0.2 μm, Merck-Millipore). For the immunodetection of S100A8 and S100A9, EPR3554 (Abcam) and monoclonal B-5 (Santa Cruz) antibodies were used. The antibodies anti-Actin (Merck millipore, clone C4) and anti-GAPDH (Life Technologies, clone 6C5) were used to detect the loading controls. Anti-rabbit or anti-mouse coupled with IRDye 800 CW or IRDye 680 (LI-COR Bioscience) secondary antibodies were used for fluorescent Western blot detection with the Odyssey Infrared Imaging system (LI-COR Biosciences).

### Stable knockdown of S100A9

Knockdown of S100A9 was achieved by shRNA interference as previously produced in our laboratory and described in our previous publication (10).

### Viral transduction of miRNA in dHL-60 cells

Optimization of suitable promoter (mEF1αpromoter) was performed using SMARTchoice Promoter Selection Plate from Dharmacon (Horizon Discovery Ltd). Briefly, 96-well plates were pre-coated with different concentrations of virus containing the turbo GFP (tGFP) under the control of seven different promoters (hCMV, mCMV, hEF1alpha, mEF1alpha, CAG, PGK or UBC). HL-60 cells were seeded in each well and over a period of ten days, GFP signals in the cells were observed by microscopy in order to determine which promoter was the most active in HL-60 cells. Lentiviral plasmids for miRNA overexpression were purchased from Dharmacon. In these plasmids, miR-132-3p (GSH11926-213626505) and miR-132-5p (GSH11926-213611344) are constitutively expressed under the control of an EF-1 alpha promoter. All constructions contain tGFP as a reporter and puromycin resistance genes.

Virus production was performed by using the “Lenti-X Packaging Single Shots (VSV-G)” kit from Takara Bio according to the manufacturer’s instructions. Briefly, HEK 293T cells were seeded at 100.000 cells per cm² in fibronectin coated Petri dishes. After 24h, plasmids were mixed with Lenti-X Packaging Single Shots tubes and dispensed on HEK 293T cells. The virus-containing medium was harvested 72h after the transfection and subsequently cleaned by centrifugation at 3.000 g and filtration (0.45 µm Millipore). The virus-containing medium was overlaid on a sucrose-containing buffer (10% sucrose m/v, 50 mM Tris-HCl, pH 7.4,100 mM NaCl, 0.5 mM EDTA) (37). Finally, viruses were resuspended in Hank’s salt solution without Ca^2+^ and Mg^2+^ (Sigma-Aldrich) and titrated using Lenti-X™ GoStix™ Plus (Clone-tech) according to the manufacturer’s recommendations.

For HL-60 infection, a 24-well plate was coated overnight at 4°C with citrate dextrose form A (ACD-A) solution containing 20 µg/mL RetroNectin. The 24-well plate was washed once with ACD-A solution and loaded with virus (~10^7^ IFU) supplemented with 6 µg/mL of DEAE-dextran. The plates were then centrifuged at 2.000 g for 2h at 32°C (38). HL-60 cells were infected at a multiplicity of infection (MOI) of 100. Thus, 100 µL culture media containing 0.1×10^6^ cells and 6 µg/mL of DEAE-dextran was added to each well, followed by centrifugation at 300 g for 5 min and incubation at 37°C with 5% CO_2_ for a minimum of 6h. Then, the media volume was adjusted, and cells were cultured for 48h. To estimate the transduction efficiency, cells were visualized with a microscope. After 14 days of selection with 0.5 µg/mL puromycin, non-clonal HL-60 sub-population overexpressing our transgene of interest was obtained.

### Statistical analysis

Statistical analysis was conducted using GraphPad Prism 8.0 software. For time course experiments involving multiple stimulatory conditions, we initiated the analysis with a two-way ANOVA, followed by a Tukey multiple comparison test. Two-group comparisons underwent preliminary assessments for normality and homogeneity of variances using Kolmogorov-Smirnov and F-tests, respectively. If the data exhibited a normal distribution and homogeneity of variances, Student’s t-test analyses were employed. Alternatively, for non-normally distributed data, Mann-Whitney tests were used for two-group comparisons. Statistical significance was determined at a p-value < 0.05.

## Results

**Figure 1:**
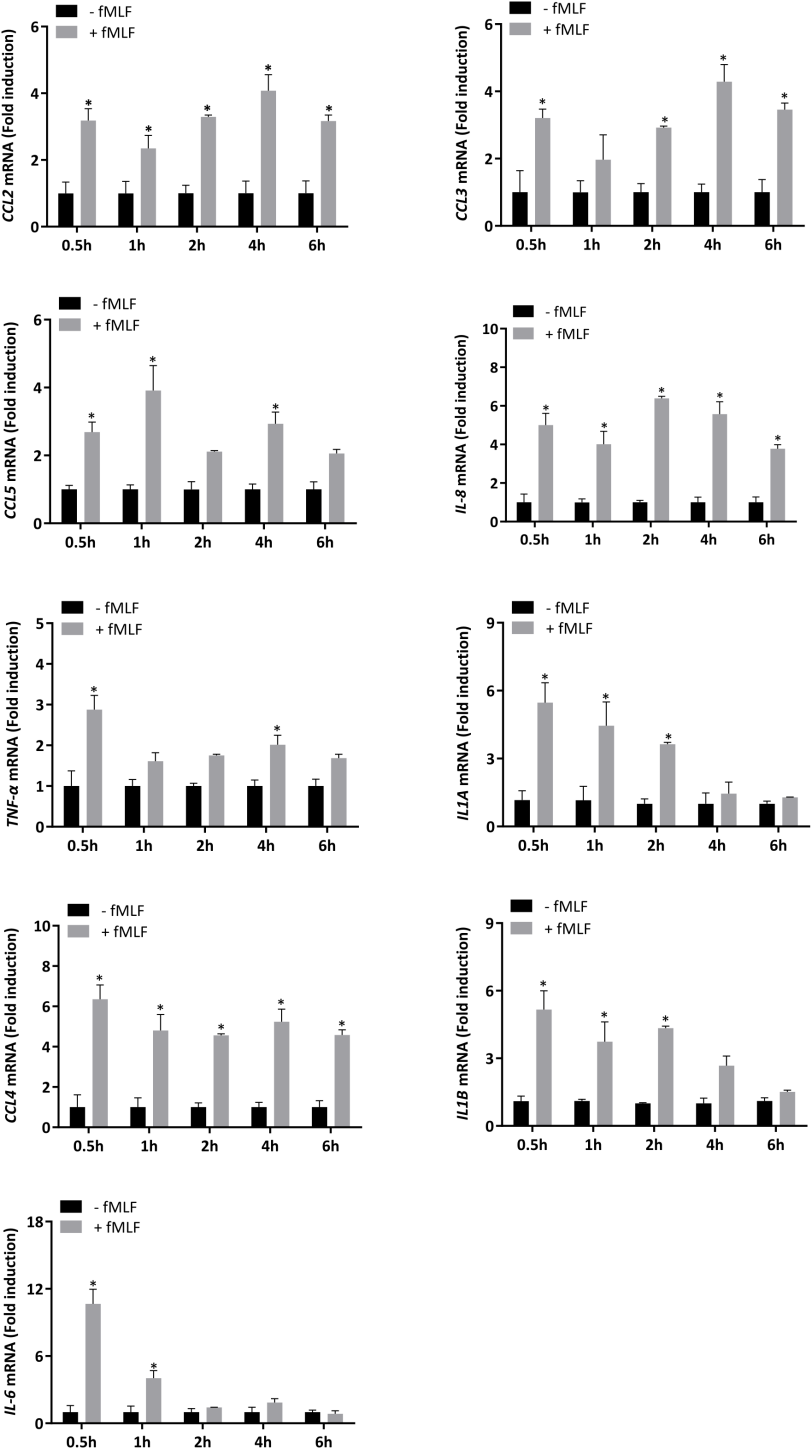
mRNA expression of cytokines stimulated by fMLF in dHL-60 cells. dHL-60 cells were stimulated with fMLF (100 nM) for 0.5h, 1h, 2h, 4h, and 6h. mRNA expression of each cytokine was assessed by RT-qPCR. Data normalization was performed by using reference genes (*Actin-β*, *B2M*, and *Gusβ*). Results are expressed as fold induction compared with control (unstimulated) cells (n = 4 biological replicates). Data are presented as mean ± SEM; *p < 0.05.

IL-8 secretion is induced by fMLF. Currently, discrepancies continue to persist in the literature on the nature of cytokine released from neutrophils according to the stimulus used. Moreover, a vast majority of studies are based on mouse models, which is now known to show different cytokine secretion patterns compared to those in human neutrophils in response to the same stimulus. In this regard, first, we investigated gene expression of several cytokines (*CCL2*, *CCL3*, *CCL4*, *CCL5*, *IL-8*, *IL-6*, *TNF-α*, *IL1A*, and *IL1B*), which have been reported to have a pro-inflammatory role (39, 40), in neutrophil-differentiated HL-60 cells. The kinetics of transcript expression indicated that each cytokine was fast upregulated after treatment with fMLF. This increased level of expression was maintained over time except for IL-6, IL1A and IL1B for which a decrease of RNA expression was observed after the peak of expression (**Figure 1**).

**Figure 2:**
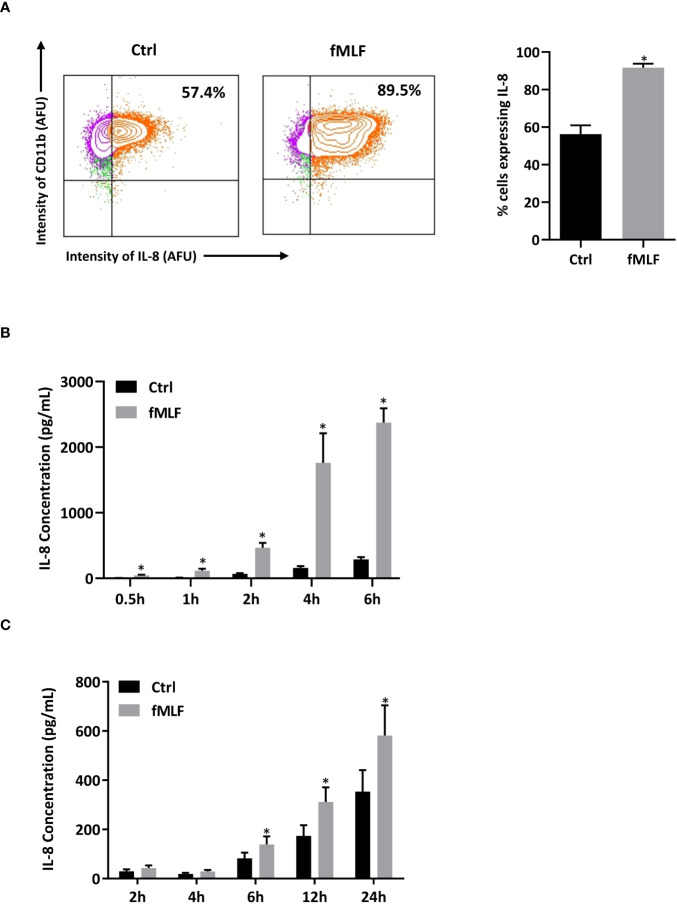
Intracellular IL-8 production and secretion after fMLF stimulation. **(A)** Cells were collected after fMLF (100 nM) stimulation for 6h. Intracellular IL-8 production was assessed by flow cytometry. Cells were gated based on single, living, differentiated and IL-8 positive gating strategy. CD11b was used as a cell surface marker to discriminate differentiated and non-differentiated HL-60 cells. Representative plots and bar graphs were shown. Cell viability was assessed by using Live/Dead™ staining followed by flow cytometry detection (insert) (n = 3 biological replicates). **(B, C)** IL-8 secretion was assessed by ELISA in **(B)** dHL-60 cells and **(C)** human neutrophils purified from peripheral blood after stimulation with fMLF (100 nM) (n = 5 biological replicates). Data are presented as mean ± SEM; *p < 0.05..

Since mRNA expression does not necessarily correlate with the level of protein (39), we performed intracellular cytokine staining to ensure that differentially expressed mRNAs correspond to an increase in cytokine production upon fMLF stimulation. Unfortunately, due to the lack of reliable antibodies able to specifically discriminate the different cytokines in neutrophils, only intracellular IL-8 was detectable in our experimental conditions. In the absence of stimulation, a significant percentage (57.4%) of total dHL-60 cells producing IL-8 was observed indicating that a pool of pre-stored IL-8 is available to be rapidly mobilizable. The percentage of positive cells for IL-8 staining was largely increased after fMLF stimulation (89.5%), showing that IL-8 *de novo* synthesis occurred (**Figure 2A**).

To confirm that IL-8 is rapidly secreted under inflammatory conditions, IL-8 present in the extracellular medium was quantified. As expected, IL-8 was found abundantly present in the supernatant after short-term fMLF stimulation, and the proportion of secreted IL-8 increased overtime (**Figure 2B**). Since we use dHL-60 cells as neutrophil models to study the mechanism of cytokine secretion, the physiological relevance of the results obtained in these cells needs to be checked. In a previous study (41), fMLF-induced IL-8 secretion profile has already been established on human neutrophils isolated from peripheral blood. However, in this work, the purity of neutrophil preparation, controlled by May-Grunwald Giemsa staining, was ≥95%. Since a low contamination of mononuclear cells (~1%) has been reported to potentially modify the cytokine expression profile attributed to neutrophils (42), we established a kinetics of IL-8 secretion after fMLF stimulation from a population of highly purified neutrophils. Although the amount of secretion was lower and delayed (**Figure 2C**) compared to dHL-60 cells, purified neutrophils showed a similar profile of IL-8 secretion validating dHL-60 cells as an appropriate cell model for our study.

### S100A8/A9 regulates IL-8 secretion in a Ca^2+^-dependent manner

**Figure 3:**
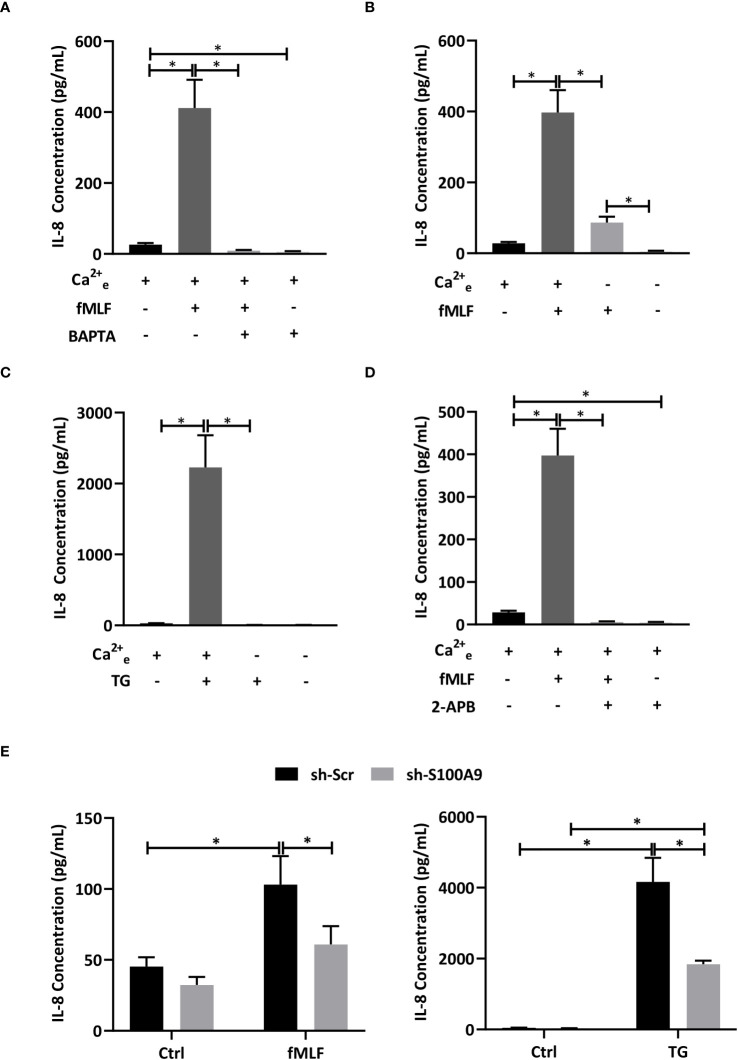
Influence of Ca^2+^ mobilization and S100A8/A9 on IL-8 secretion. **(A)** dHL-60 cells were incubated with BAPTA/AM (10 μM) for 30 min before stimulation with fMLF (100 nM) for 6h (n = 6 biological replicates). **(B)** Cells were stimulated with fMLF (100 nM) for 6h in the presence or absence of extracellular Ca^2+^ (n = 6 biological replicates). **(C)** Cells were stimulated with thapsigargin (TG, 100 nM) for 6h in the presence or absence of extracellular Ca^2+^ (n = 6 biological replicates). **(D)** Cells were incubated with 2-APB (10 μM) for 30 min before stimulation with fMLF (100 nM) for 6h (n = 6 biological replicates). **(E)** Cells containing stably integrated shRNA-S100A9 (sh-S100A9), or shRNA-Scramble were stimulated with fMLF (100 nM) or thapsigargin (TG, 100nM) for 6h (n = 4 biological replicates). Secretion of IL-8 was assessed by ELISA. Data are presented as mean ± SEM; *p < 0.05..

In an elegant study, Clemens et al. (7) concluded that store-operated Ca^2+^ entry was involved in the regulation of cytokine secretion in mouse neutrophils. However, we are now aware that results obtained in mouse neutrophils need to be carefully translated to human neutrophils since major differences exist between both species notably at the immunological level (43). In this regard, IL-8 has never been identified in mice. In this context, we investigated the importance of Ca^2+^ signaling on fMLF-induced IL-8 secretion. First, dHL-60 cells were incubated with BAPTA, an intracellular Ca^2+^ chelator. fMLF-induced IL-8 secretion was totally inhibited in the presence of BAPTA (**Figure 3A**) indicating that intracellular Ca^2+^ signaling is absolutely required for IL-8 secretion. In neutrophils, changes in intracellular Ca^2+^ concentration [Ca^2+^]_i_ can be mediated by either an extracellular Ca^2+^ entry or Ca^2+^ release from intracellular stores. To determine the role of Ca^2+^ influx resulting from an extracellular Ca^2+^ entry, dHL-60 were stimulated with fMLF in the presence or absence of extracellular Ca^2+^. As expected, removal of extracellular Ca^2+^ resulted in a drastic reduction of IL-8 secretion (**Figure 3B**). To note, while IL-8 was totally abolished by BAPTA (**Figure 3A**), removal of extracellular Ca^2+^ allowed a slight increase of fMLF-induced IL-8 secretion (**Figure 3B**) suggesting that Ca^2+^ release from intracellular stores could be sufficient to mobilize IL-8 or/and a second signal, independent of Ca^2+^, intervene in IL-8 secretion. To confirm the importance of Ca^2+^ entry on IL-8 secretion, thapsigargin was used to mediate Ca^2+^ influx through SOCE without triggering receptor-ligand interactions (44). Thapsigargin was able to mediate a much higher IL-8 secretion than fMLF. In the absence of extracellular Ca^2+^, thapsigargin-induced IL-8 secretion was totally inhibited (**Figure 3C**) confirming that extracellular Ca^2+^ is absolutely necessary for IL-8 secretion. Finally, we used 2-APB, which was largely used to inhibit SOCE at a concentration range of 10-50 μM (45). Our data indicated that IL-8 secretion stimulated by fMLF were totally inhibited when dHL-60 cells were pre-treated with 2-APB (**Figure 3D**).

Taken all together, these results indicate that an extracellular Ca^2+^ entry mediated by SOCE is absolutely required for fMLF-induced IL-8 secretion in neutrophil-like HL-60 cells. However, the question arises of how the Ca^2+^ response can be transduced into IL-8 secretion. In a previous work, we suggested that Ca^2+^- and Zn^2+^-binding proteins S100A8/A9 could be involved in cytokine secretion mediated by LPS as Toll-like receptor 4 agonist (10). However, it is still not known if S100A8/A9 can also act as key regulators of IL-8 secretion induced by intracellular signaling pathways induced by G protein-coupled receptors. Stable knockdown of S100A8/A9 by shRNA provoked a reduction of IL-8 secretion mediated by fMLF and thapsigargin (**Figure 3E**) arguing for an essential role of S100A8/A9 for IL-8 secretion through SOCE.

Furthermore, it has been reported that SOCE could regulate NF-κB activation to support cytokine synthesis (7). Thus, we postulate that the Ca^2+^-dependent release of IL-8 is modulated through S100A8/A9 which activate NF-κB allowing *de novo* synthesis of IL-8 and an increase in the pool able to be secreted.

**Figure 4:**
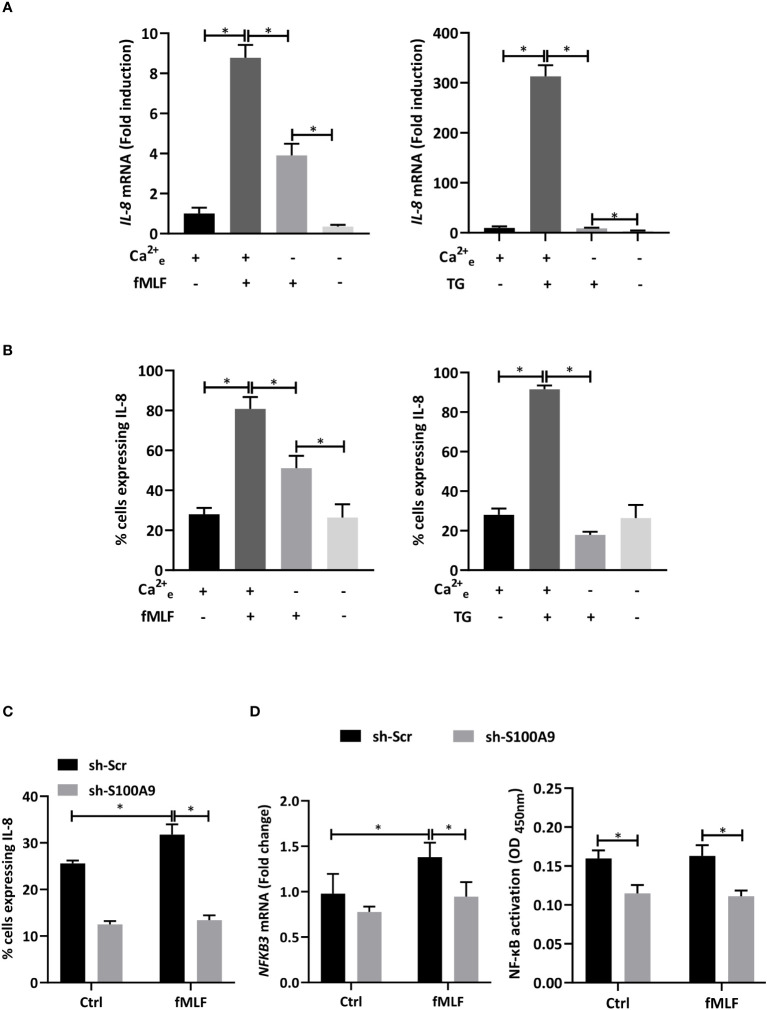
IL-8 production is dependent on S100A8/A9-dependent NF-κB activation. **(A, B)** dHL-60 cells were collected after stimulation with fMLF (100 nM) or thapsigargin (TG, 100 nM) for 6h in the presence or absence of extracellular Ca^2+^. **(A)** The expression of IL-8 was assessed by RT-qPCR. Data normalization was performed using reference genes (*Actin-β*, *B2M*, and *Gusβ*). Results are expressed as fold induction compared with control (unstimulated) cells (n = 6 biological replicates) **(B)** Intracellular IL-8 production was assessed by flow cytometry analysis. Cells were gated based on single, living, differentiated and IL-8 positive gating strategy (n = 3 biological replicates). **(C, D)** Cells with shRNA-S100A9 (sh-S100A9) or shRNA-Scramble (sh-Scr) were stimulated with fMLF (100 nM) for 6h. **(C)** Intracellular IL-8 production was assessed by flow cytometry analysis (n = 4 biological replicates). **(D)** *NFκB3* expression was assessed by RT-qPCR (left panel). Data normalization was performed in using reference genes (*Actin-β*, *B2M*, and *Gusβ*). Results are expressed as fold induction compared with control (unstimulated) cells (n = 5 biological replicates). NF-κB activation assay was performed (n = 3 biological replicates). Data are presented as mean ± SEM; *p < 0.05.

In accordance with this hypothesis, IL-8 mRNA expression (**Figure 4A**) and positive cells for IL-8 intracellular staining (**Figure 4B**) induced by fMLF and thapsigargin were strongly reduced in the absence of extracellular Ca^2+^. Then, a stable knockdown of S100A9 was performed to determine the impact of S100A8/A9 on IL-8 production. Indeed, absence of S100A9 is well-described to trigger an extinction of both S100A8 and S100A9 (46) as previously checked in our experimental conditions (10). As expected, inhibition of S100A9 provoked a decrease in IL-8 production (**Figure 4C**). Moreover, depletion of S100A8/A9 was able to inhibit *NFκB3* mRNA expression and NF-κB activation (**Figure 4D**). However, unexpectedly, fMLF was not able to increase NF-κB-activity. Taken together, these data allow to conclude that an extracellular Ca^2+^ entry controls IL-8 secretion through the regulation of NF-κB-activated S100A8/A9 but other signalling pathways are likely involved in this regulation.

### miR-132-5p negatively regulates S100A8 and S100A9 expression

Growing evidence shows that miRNAs participate in the regulation of various neutrophil pro-inflammatory functions (47, 48), notably miR-146a-5p and miR-155-5p, which are involved in a negative feedback loop of cytokine secretion (29). Based on these findings, we performed a network analysis to identify potential miRNAs involved in the regulation of S100A8/A9 expression and thus, potentially in IL-8 secretion.

**Figure 5:**
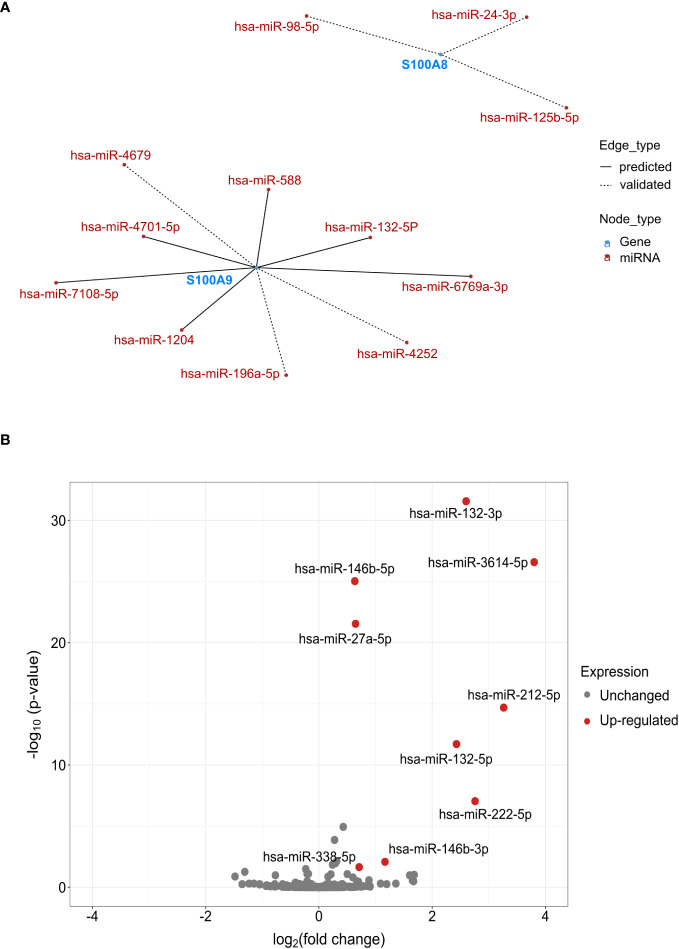
Potential miRNAs regulating S100A8 and S100A9. **(A)** Network analysis of miRNAs regulating S100A8 and S100A9. Analysis was performed using multiMiR package based on the data from miRTarBase 8.0 and TargetScan 7.2. The top 35% miRNAs were selected as potential targets. **(B)** Volcano plot represented the -log10 p-values (y-axis) *versus* the log2 FC (Fold Change) (x-axis) for each miRNA after fMLF (100 nM) stimulation for 6h. Only miRNAs fulfilled the criteria of FDR < 5% and log2 FC ≥ 0.5 were labeled in red in the volcano plot. All the analysis for **(A, B)** was done using R program.

Based on data from miRTarBase and TargetScan (**Figure 5A**), three and nine miRNAs were predicted or validated to target S100A8 and S100A9, respectively. To validate if these miRNAs could be involved in the regulation of S100A8 and S100A9 expression, we performed miRNA sequencing in dHL-60 cells stimulated with fMLF for 6h. Volcano plots of the differentially expressed (DE) miRNAs showed that nine miRNAs were upregulated including miR-132-5p (**Figure 5B**), which was previously predicted to target S100A9, and miR-132-3p that could target the same transcripts as miR-132-5p (49). In this context, both species of miR-132 were examined for their eventual role in S100A8/A9-dependent IL-8 secretion.

**Figure 6:**
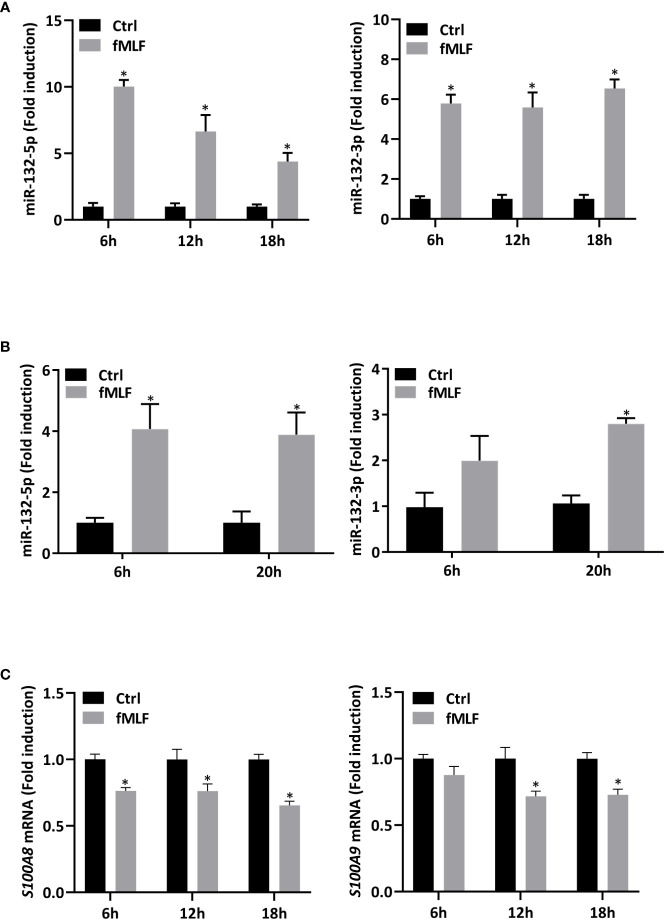
Expression of miR-132 and S100A8/A9 are dysregulated upon fMLF stimulation. **(A)** dHL-60 cells were stimulated with fMLF (100 nM) for 6h, 12h, and 18h. Expression of miR-132-3p and miR-132-5p were assessed by RT-qPCR. Data normalization was performed using three reference genes (SNORD44, miR-103a-3p, SNORD38B). Results are expressed as fold induction compared with control (unstimulated) cells (n = 4 biological replicates). **(B)** Human neutrophils were stimulated with fMLF (100 nM) for 6h and 20h. Expression of miR-132-3p and miR-132-5p were assessed by RT-qPCR. Data normalization was performed using SNORD44. Results are expressed as fold induction compared with control (unstimulated) cells (n = 4 biological replicates). **(C)** dHL-60 cells were stimulated with fMLF (100 nM) for 6h, 12h, and 18h. Expression of S100A8 and S100A9 were assessed by qPCR. Data normalization was performed using three reference genes (*Actin-β*, *B2M*, and *Gusβ*). Results are expressed as fold induction compared with control (unstimulated) cells (n = 4 biological replicates). Data are presented as mean ± SEM; *p < 0.05.

**Figure 7:**
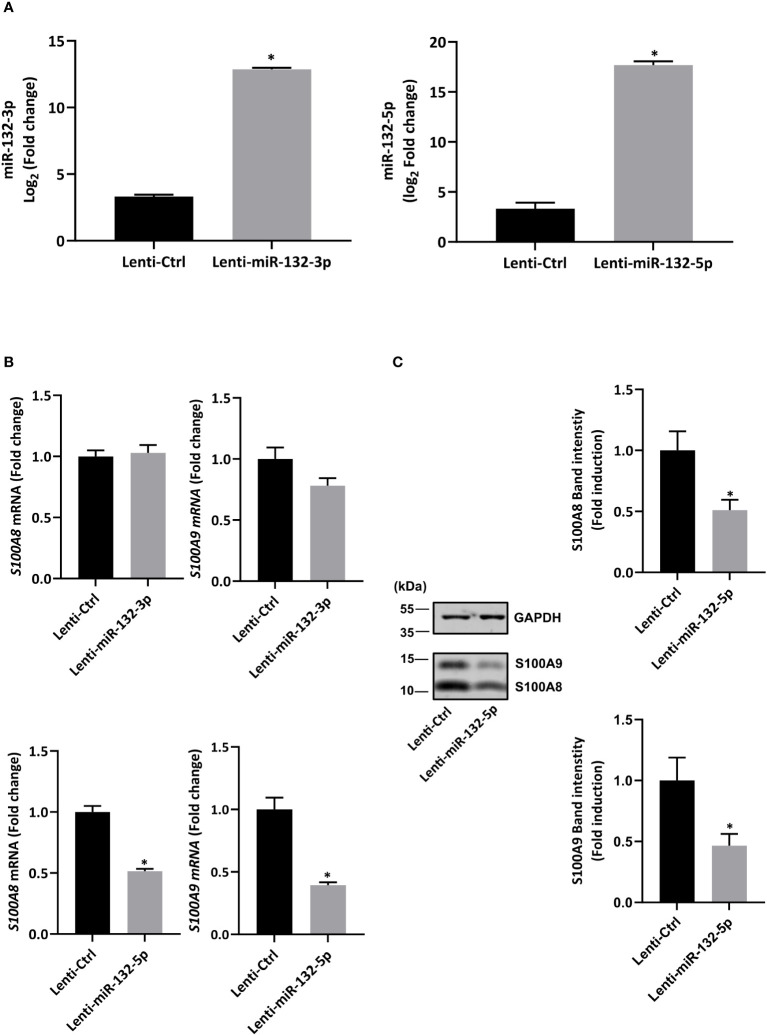
Stable overexpression of miR-132-5p decreases S100A8/A9 expression in resting dHL-60 cells. **(A)** Expression of miR-132-3p and miR-132-5p were measured by RT-qPCR in transduced dHL-60 cells. Data normalization was performed using three reference genes (SNORD44, miR-103a-3p, SNORD38B). Results are expressed as fold induction compared with transduced negative control (n = 6 biological replicates). **(B)** Expression of S100A8 and S100A9 in transduced dHL-60 cells with miR-132-3p and miR-132-5p was detected by RT-qPCR. Data normalization was performed using three reference genes (*Actin-β*, *B2M*, and *Gusβ*). Results are expressed as fold induction compared with transduced negative control (n = 6 biological replicates). **(C)** Expression of S100A8 and S100A9 protein in transduced dHL-60 cells with miR-132-5p was detected by Western blot. A representative Western blot and relative densitometric bar graphs are shown (n = 5 biological replicates). The band intensity of S100A8 and S100A9 were normalized to glyceraldehyde-3-phosphate dehydrogenase (GAPDH). Data are presented as mean ± SEM; *p < 0.05..

**Figure 8:**
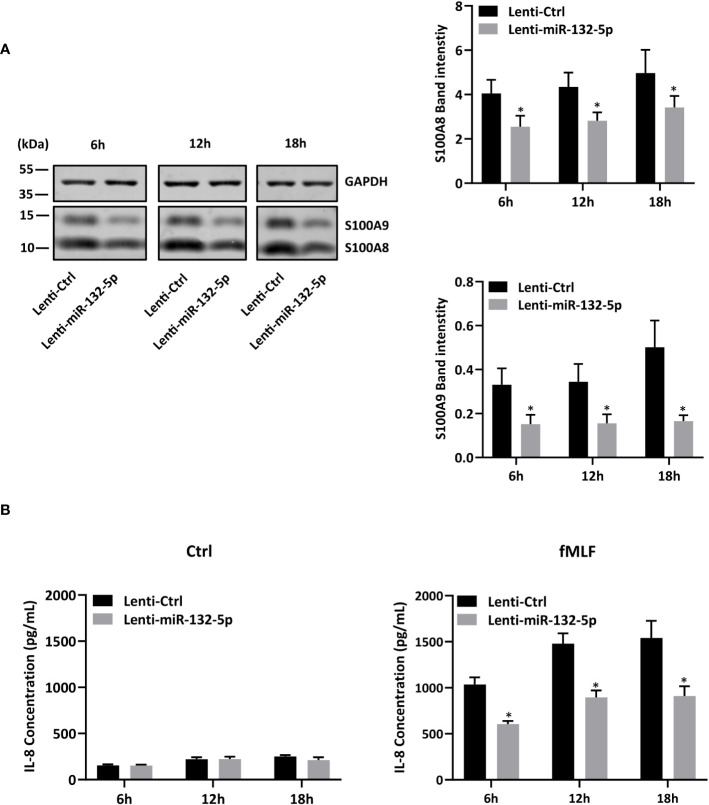
miR-132-5p regulates IL-8 secretion through S100A8/A9. **(A, B)** Transduced dHL-60 cells with miR-132-5p were stimulated with fMLF (100 nM) for 6h, 12h and 18h. **(A)** Expression of S100A8 and S100A9 proteins in transduced dHL-60 cells with miR-132-5p were detected by Western blot. A representative Western blot and relative densitometric bar graphs are shown. The band intensities of S100A8 and S100A9 were normalized to GAPDH (n = 5 biological replicates). **(B)** Secretion of IL-8 secretion was measured by ELISA (n = 5 biological replicates). Data are presented as mean ± SEM; *p < 0.05..

**Figure 9:**
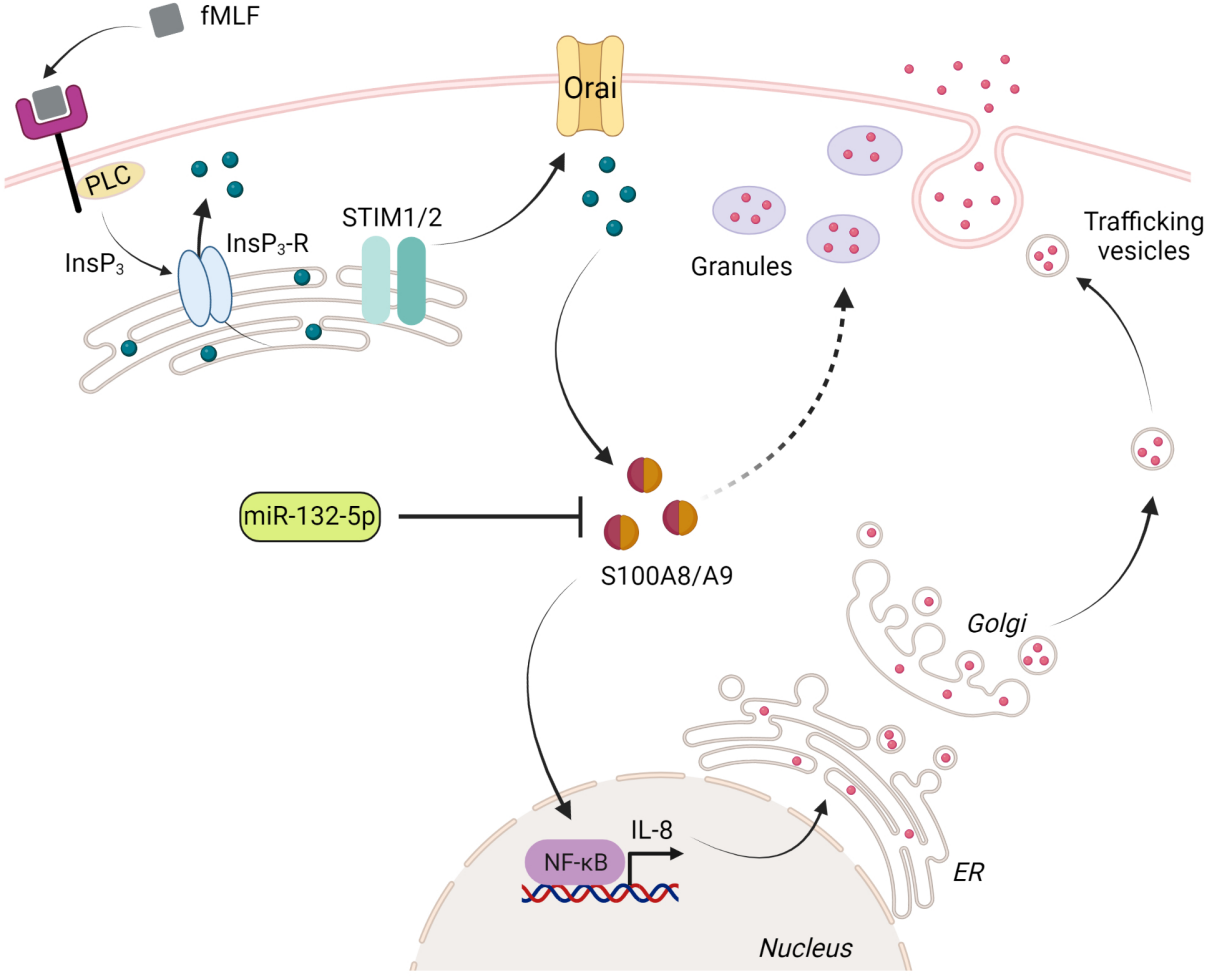
Proposed model for the regulation of IL-8 secretion by S100A8/A9 and miR-132-5p. Upon stimulation of fMLF, phospholipase C (PLC) induces inositol 1,4,5-trisphosphate (InsP_3_), which binds to its receptor (InsP_3_-R) localized in the endoplasmic reticulum (ER). The fall of Ca^2+^ level in the ER is sensed by STIM1 or/and STIM2, which mediates the activation of store-operated Ca^2+^ channels (Orai) in the plasma membrane. Orai activation leads to a sustained elevation of cytosolic Ca^2+^ concentration. S100A8/A9 binds Ca^2+^ and ensures the regulation of IL-8 secretion through NF-κB activation. Preformed IL-8 or *de novo* synthesized IL-8 can be released through the degranulation process (regulated exocytosis) or trafficking vesicles (constitutive exocytosis), respectively. The excessive release of IL-8 is impaired by a negative regulation of miR-132-5p on S100A8/A9 expression. The figure was created using Biorender (https://biorender.com).

First, in order to validate RNA-seq results, expression of miR-132-3p and -5p was quantified after fMLF stimulation for 6h and more. While the increase of miR-132-3p expression was similar at each time point (**Figure 6A**, right panel), fMLF-induced miR-132-5p expression was decreased overtime (**Figure 6A**, left panel). In addition, miR-132-3p and -5p expression were validated in human neutrophils. As observed in dHL-60 cells, fMLF was able to increase the expression of both miRNAs albeit at a lower level (**Figure 6B**). Next, we investigated if an increase in miR-132 expression was correlated with a decrease of S100A8 and S100A9 expression. As expected, levels of S100A8 and S100A9 were dysregulated when dHL-60 cells were stimulated by fMLF (**Figure 6C**). To further support our assumption that miR-132 can negatively regulate S100A8/A9, triggering a decrease of IL-8 secretion, we transfected HL-60 cells with lentiviral particles to induce a stable overexpression of miR-132-3p or miR-132-5p. Quantification analysis by qPCR showed a logarithmic increase of miR-132-3p and miR-132-5p expression indicating that successful overexpression of both miRNAs in dHL-60 cells was achieved (**Figure 7A**). Then, the expression of *S100A8* and *S100A9* was determined in the knockdown cell lines at the resting state. Overexpression of miR-132-3p was not able to impact *S100A8* and *S100A9* mRNA expression (**Figure 7B** **).** On the contrary, *S100A8* and *S100A9* expression was largely reduced when miR-132-5p was overexpressed (**Figure 7B**). The decrease of S100A8 and S100A9 after overexpression of miR-132-5p was confirmed at the protein level (**Figure 7C**). miR-132-5p overexpression was also able to inhibit S100A8 and S100A9 in an inflammatory context, when lenti-miR-132-5p cells were stimulated with fMLF. Protein expression of S100A8 and S100A9 was reduced during prolonged stimulation (**Figure 8A**). Finally, the role of miR-132-5p in the regulation of IL-8 secretion was investigated. Overexpression of miR-132-5p in dHL-60 was able to inhibit IL-8 mediated by fMLF (**Figure 8B**). Collectively, these results clearly demonstrate for the first time that miR-132-5p regulates IL-8 secretion through the downregulation of S100A8/A9 expression (**Figure 9**).

## Discussion

The accumulation of indisputable evidence has highlighted that neutrophils are involved in the pathogenesis of chronic inflammatory and autoimmune diseases. More recently, their preponderant roles, through the secretion of cytokines, in the initiation and progression of tumors have been underlined (50, 51). While the influence of cytokines on intracellular signaling pathways on target cells has been largely investigated, mechanisms surrounding cytokine secretion in neutrophils are poorly defined and even the nature of cytokine secreted is still open to debate. The diversity of stimuli used, the difference observed in the cytokine pattern between mice and humans coupled with the challenge represented to manipulate neutrophils are probably the cause of this lack of consensus. In this context, we propose to explore mechanisms related to Ca^2+^-dependent cytokine secretion in human neutrophil-like cells.

### Cytokine mobilization in neutrophils

Stimulation of differentiated HL-60 cells with a pro-inflammatory stimulus such as fMLF, was able to increase the expression of a large range of cytokines. However, all cytokines found expressed at the transcriptional level were not necessarily detected in the extracellular medium (data not shown). Indeed, an accumulation of intracellular mRNAs could be not sufficient to support their production and subsequent release. Moreover, the secretion of these cytokines could be relatively low and therefore not detectable in our experimental conditions. Finally, fMLF could be not able to activate appropriate signalling pathways necessary for the release of these cytokines. Indeed, a combination of several pathways can take place in neutrophils to ensure an optimal delivery of cytokines during the inflammation process. In this view, secretion of IL-1α and IL-1β has been reported to occur via the non-canonical pathway due to their lack of signal sequences. In addition, IL-1β has been suggested to be released through plasma transporters (52) or unconventional autophagy (53). This could explain the fact that IL-1α and IL-1β mRNA expression is increased (data not shown) but not secreted upon fMLF stimulation.

Among the cytokines strongly released, IL-8 was found to be stored as a pool of preformed cytokines able to be rapidly mobilizable during inflammation. IL-8 was *de novo* synthesized upon fMLF stimulation to provide a sufficient quantity of IL-8 into the extracellular environment to ensure an efficient pro-inflammatory response. Cytokines could be secreted through the degranulation process (54, 55), which has been described to be regulated by an increase of intracellular Ca^2+^ (56, 57). Thus, since fMLF is known to mediate granule release in neutrophils (58), we cannot exclude that fMLF controls the release of preformed IL-8 through the regulation of degranulation. To support this idea, it has been previously predicted, based on a linear fitting approach, that IL-8 can be stored in secretory vesicles and also in gelatinase and specific granules (55).

### Role of Ca^2+^ signalling in cytokine secretion

Our results showed that extracellular Ca^2+^ entry through SOCE is a key regulator of IL-8 secretion. However, the contribution of Ca^2+^ signaling could be dependent on the type of cytokine secreted since our data indicate that Ca^2+^ mobilization is absolutely required but not sufficient for an optimal IL-8 secretion while secretion of CCL2, CCL3, CCL4 was totally dependent on Ca^2+^ changes (data not shown). Convincing results support the role of STIM proteins in the regulation of cytokine secretion in mice. A reduction of TNF-α and IL-6 levels in the peritoneum of mice with neutrophil STIM2 ablation prevented the deleterious effects caused by a systemic inflammatory response (7). In the same way, no alteration of imiquimod-induced cytokine expression was reported in mice lacking functional STIM1 in a context of psoriasis-inflamed skin (8). These two studies excluded a contribution of STIM1 in cytokine secretion and highlighted a role for the STIM2 isoform. However, more recently, Kahlfuss et al. (59), reported that a suppression of IL-17A in human non-pathogenic Th17 cells ablated for STIM1. Therefore, the contribution of STIM1 or/and STIM2 to cytokine production could be either species-or/and cell-specific. The role of STIM isoforms needs to be carefully examined in human neutrophils before concluding their roles in cytokine secretion.

### Intracellular S100A8/A9 are key regulators of IL-8 secretion

As a result of an increase in [Ca^2+^]_i_, Ca^2+^-binding proteins S100A8/A9 have been involved to transduce the inflammatory responses notably through the regulation of NADPH oxidase activity (25). Our present data supported by our previous work (10), provide evidence that S100A8/A9 can constitute a common node for signaling pathways mediating IL-8 secretion induced by different types of stimuli through the regulation of NF-κB activation. S100A9 can be phosphorylated on its Thr113 by p38 MAPK upon fMLF stimulation (60) and the phosphorylated form of S100A8/A9 has been reported to be functionally relevant (21, 23). In this sense, intracellular phosphorylated S100A8/A9 has been demonstrated to be essential for an optimal NADPH oxidase activity (25). Further studies are necessary to investigate whether S100A9 phosphorylation is required for an active involvement of S100A8/A9 in cytokine secretion. This would allow us to define whether S100A9 phosphorylation can constitute a level of regulation to modulate neutrophil cytokine secretion.

### miRNAs negatively regulate fMLF-induced IL-8 secretion

Since S100A8/A9 are abundant in resting neutrophils and possess a preponderant role in the amplification of pro-inflammatory responses, the question is raised of how S100A8/A9 could be regulated in order to prevent an exacerbation of inflammation. Growing evidence over the years shows that miRNAs allow a fine-tuning of neutrophil pro-inflammatory functions (61) and can control the overall severity of the inflammatory response through negative feedback loops (30). Indeed, miRNAs can accumulate overtime within the cells and impair an excessive inflammatory response when inflammation is prolonged. In this context, we established that overexpression of miR-132-5p can decrease S100A8/A9 expression level resulting in a reduction of IL-8 secretion. In a monocytic cell line, THP-1, stimulated by LPS, was also able to induce miR-132 expression (28), however, the functional relevance of such an observation was not investigated. The upregulation of miRNA-132 has been associated with T cell activation (62). In this study, miR-132 deficiency in CD4^+^ T cells of mice with chronic infection provoked a higher and lower levels of IL-10 and IFNγ production, respectively, compared to wild-type cells. miR-132 knockout resulted in an enhancement of susceptibility to pathogens and an alteration of the protective response generated by inflammation.

Taken together, these data highlight the critical role of miR-132 in the inflammation and immunity. We provide clear evidence on the fact that dysregulation of miRNA-132-5p expression in neutrophil-like cells can modulate the intensity of inflammation and prevent its persistence of inflammation by acting on S100A8/A9-mediated IL-8 secretion (**Figure 9**). Further investigations are still required in the different immune cell types to determine whether miR-132 can act as a pro- or anti-inflammatory regulator and characterize the underlying mechanisms.

## Conclusion

Given its potential role in the modulation of immune responses, miR-132-5p could represent an important tool in the treatment of inflammation disorders especially in light of recent advances in the delivery of miRNAs through packaging in extracellular vesicles and transfer to target inflamed cells or tissue to facilitate the transition from a pro-inflammatory towards an anti-inflammatory state. Moreover, given the importance of cytokine mobilization from a clinical perspective, considerable efforts still need to be devoted to understanding mechanisms triggering cytokine secretion in order to potentially improve treatment or design curative treatments. Given the complexity of the mechanism involved, the lack of appropriate tools, the difficulty of genetically modifying primary neutrophils and the use of models not faithfully recapitulating the complete phenotypic and functional properties of neutrophils, such studies remain challenging. Overcoming these limitations is absolutely necessary since a profound knowledge on neutrophil biology holds the promise to improve current therapy through the modulation of specific cytokine release and the reduction of adverse effects associated to these therapies.

## Data availability statement

The datasets presented in this study can be found in online repositories. The names of the repository/repositories and accession number(s) can be found below: https://www.ebi.ac.uk/ena, PRJEB64660.

## Ethics statement

The studies involving humans were approved by the Ethics Review Panel (ERP) of the University of Luxembourg based on the guidelines of the “Comité National d’Ethique de Recherche” (CNER) from Luxembourg. The studies were conducted in accordance with the local legislation and institutional requirements. The participants provided their written informed consent to participate in this study.

## Author contributions

SB: Conceptualization, Investigation, Methodology, Supervision, Writing – original draft. YZ: Data curation, Formal analysis, Investigation, Methodology, Validation, Writing – original draft. MT: Formal analysis, Validation, Writing – review & editing. AG: Formal analysis, Writing – review & editing. FT: Writing – review & editing, Investigation, Methodology. HLW: Methodology, Writing – review & editing. J-LB: Writing – review & editing, Project administration, Supervision.
